# Uterine wall rupture in a primigravid patient with oligohydramnios as the first manifestation: A case report

**DOI:** 10.1097/MD.0000000000024051

**Published:** 2021-01-15

**Authors:** Lingyun Yang, Bo Zhang, Yifan Zhao, Chuan Xie

**Affiliations:** a Department of Gynecology and Obstetrics, West China Second University Hospital; b Key Laboratory of Birth Defects and Related Diseases of Women and Children, Ministry of Education; c Department of Ultrasound, West China Second University Hospital, Sichuan University.

**Keywords:** oligohydramnios, pregnancy, unscarred, uterine rupture

## Abstract

**Rationale:**

Spontaneous uterine rupture during pregnancy, occurring most often during labor in the context of a scarred uterus, is a serious obstetric complication. Perhaps even more serious because of its extreme rarity, spontaneous uterine rupture in a primigravid patient with an unscarred gravid uterus would be essentially unexpected. Clinical manifestations of unscarred uterine ruptures are nonspecific and can be confusing, making a correct early diagnosis very difficult.

**Patient concerns:**

A primigravid woman at 27 weeks of gestation presented to our hospital with acute oligohydramnios. Ultrasound examination at her local hospital revealed oligohydramnios that had not been present 1 week previously. A specific cause of the acute oligohydramnios, however, was not established.

**Diagnosis:**

Upon transfer to our hospital, the patient was hemodynamically stable without abdominal tenderness or peritoneal signs. Transabdominal ultrasound was repeated and confirmed oligohydramnios and seroperitoneum. The fetal heart rate was in the normal range, and blood tests revealed a low hemoglobin level of 91 g/L, which had been normal recently. A repeat sonogram after admission found that there was almost no amniotic fluid within the uterine cavity, and there was increased peritoneal fluid. Repeat hemoglobin showed a further decrease to 84 g/L. The combination of increased free abdominal fluid, lack of intrauterine fluid, and acutely decreasing hemoglobin strongly suggested uterine rupture with active intraperitoneal bleeding.

**Interventions:**

Emergent laparotomy was performed, and a male infant was delivered. Comprehensive abdominal exploration revealed a rupture in the right uterine cornua with ongoing slow bleeding, through which a portion of the amniotic sac protruded into the abdominal cavity.

**Outcomes:**

The laceration was repaired, the patient and neonate recovered without complications, and were discharged 5 days postoperatively.

**Lessons:**

An increased awareness of the rare but real possibility of spontaneous uterine rupture in a primigravid patient with no prior uterine scarring helped to establish an earlier diagnosis. Obstetricians should consider this possibility in pregnant females, even in the absence of risk factors and in early gestational age, when sudden unexplained clinical manifestations, such as acute oligohydramnios, are encountered.

## 1 Introduction

Uterine rupture (UR) during pregnancy is one of the true life-threatening complications encountered in obstetric practices.^[1]^ There are several high-risk factors associated with UR, the most common of which is a scarred uterus subsequent to prior cesarean section (CS). However, UR in the primigravid patient without any high-risk factor is extremely rare, and there are few typical clinical symptoms.^[2,3]^ Reports indicate that abdominal pain may be the principal clinical presentation during the first and second trimester of pregnancy, while fetal heart abnormalities can be the indicator during the third trimester and during labor.^[4]^ We present a case of a primigravid woman suffering UR at 27 weeks gestation, presenting with oligohydramnios as the first clinical manifestation.

## 2 Case report

Ethical approval and patient consent were acquired and recorded in the patient medical record with witness signature. All ethical approval and consent procedures were approved by the Medical Ethical Committee of West China Second University Hospital, Sichuan University.

**Figure 1:**
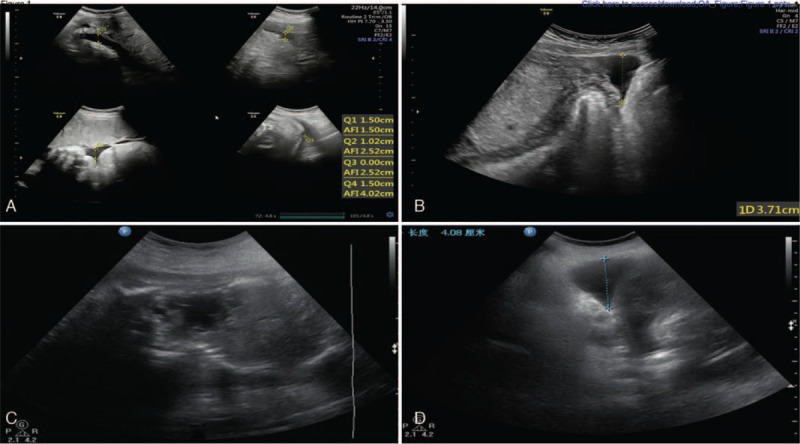
The sonographic findings of the patient with unscarred uterine rupture. The sonographic examination did not find any laceration in the uterine wall. Figure A and B were the results of transabdominal ultrasound performed on admission. Figure A demonstrated oligohydramnios; the amniotic fluid index was 4.02, and the maximum pool depth was 1.5 cm. Figure B revealed seroperitoneum; the maximum depth of abdominal effusion was 3.71 cm. Figure C and D were the results of repeated sonographic examination performed after admission. Figure C demonstrated there was almost no amniotic fluid in the amniotic cavity. Figure D revealed increased peritoneal fluid; the maximum depth of abdominal effusion is 4.08 cm.

A 35-year-old primigravid woman at 27 weeks gestation presented to her local hospital with mild abdominal discomfort. This symptom lasted for only a brief time, but led to abdominal ultrasound and oligohydramnios was found for the first time during her pregnancy, and the patient was urgently transferred to our hospital. Her pregnancy course up to that point had been uncomplicated. She reported no prior abdominal operations or trauma, and she denied vaginal discharge or hemorrhage. On physical examination, her vital signs and general examination findings were normal, specifically without abdominal tenderness or uterine contractions. The fetal heart rate was essentially normal, ranging from 144 to 162 beats per minute (bpm). A repeat ultrasound examination confirmed the presence of oligohydramnios (amniotic fluid index was 4.02, and maximum pool depth was 1.5 cm, Fig. 1A) and seroperitoneum (Fig. 1B). The hemoglobin level was 91 g/L, a drop from her normal value previously during her pregnancy. Dexamethasone was given to the mother to accelerate fetal lung maturation, and another obstetric ultrasound was performed at 27^+2^ weeks gestation. There was almost no amniotic fluid within the uterine cavity (Fig. 1C), while the seroperitoneum had increased to 4.08 cm (Fig. 1D). The hemoglobin had further decreased to 84 g/L. Interestingly, the fetal heart rate had remained in the normal range. The increased seroperitoneum and decrease in hemoglobin strongly indicated ongoing intraperitoneal bleeding, ominous for occult uterine rupture.

**Figure 2:**
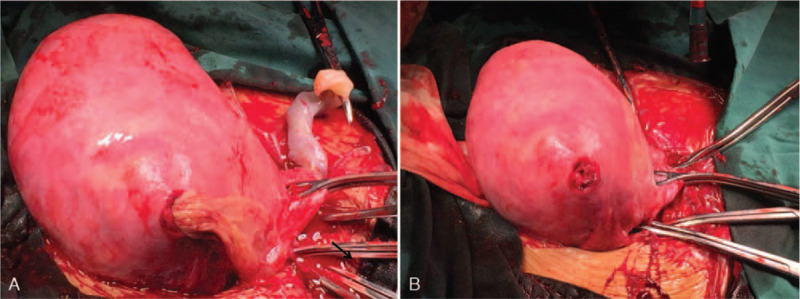
intraoperative findings of the patient with unscarred uterine rupture. Figure A showed that part of the decompressed amniotic sac protruded into the abdominal cavity through this rupture, and the amniotic fluid had slowly leaked out. The arrow in Figure A points to the amniotic membrane. Figure B revealed a rupture of approximately 1.5 cm with slow bleeding in the right uterine cornua.

Consequently, the patient was quickly moved to the operation room, where, in consultation with the obstetrical team, an emergency cesarean delivery under general anesthesia was performed. Upon entering the abdominal cavity, approximately 1000 ml of hemoperitoneum was encountered in the abdominal cavity extending into the pelvis. The size of the uterus was significantly smaller than would be expected for the 27-week gestational age. Comprehensive abdominal examination revealed a rupture of approximately 1.5 cm with slow bleeding in the right uterine cornua (Fig. 2 A and B), and part of the decompressed amniotic sac protruded into the abdominal cavity through this rupture, and the amniotic fluid had slowly leaked out (Fig. 2A). The baby was rapidly delivered within 2 minutes. The Apgar Scores were 7 and 7 at 1 and 5 minutes after birth. The baby weighed 1130 g and was transferred to the neonatal intensive care unit (NICU). After hemostasis was secured, the rupture in the uterine wall was repaired with several figure-of-eight sutures. The patient was discharged home on her 5th postpartum day without any complications. Subsequent follow-up confirmed both mother and baby were in good condition.

## 3 Discussion

Uterine rupture, occurring principally in a patient with a scarred uterus, is an uncommon yet often a severe, emergency complication of pregnancy. However, rupture of an unscarred uterus in a primigravid patient is an extremely rare event, with an estimated incidence of 1:7643 to 1:16,849 in a series from the United States.^[5]^ The consequences of this rare event, including maternal hemorrhage, perinatal death and hysterectomy, may be catastrophic. Thus, prompt recognition of uterine rupture and emergent laparotomy remain the critical factors influencing maternal and perinatal morbidity and mortality.^[6]^

The clinical signs and symptoms of uterine rupture in primigravid patients are similar to those in parous women. Classically, the clinical findings of rupture of a gravid uterus, regardless of etiology, are abdominal pain, vaginal bleeding, fetal compromise, cessation of uterine contractions, and maternal tachycardia.^[6,7]^ However, it is rare for all these clinical manifestations to be evident. Walsh et al conducted a review of published cases over a 60-years span of uterine rupture in primigravid patients. Of 36 cases, 26 presented with abdominal pain, frank hypovolemic shock was evident in 8, fetal compromise in 16, and vaginal bleeding in only 4 cases. Thirty two cases occurred during the third trimester, and only 4 in the first and second trimesters.^[8]^ Although the presenting features were similar to those in parous women, the obstetricians understandably had a lower index of suspicion for primigravid rupture. Furthermore, the clinical manifestations of uterine rupture during the first or second trimester are nonspecific and can be confusing. Hence, early diagnosis of uterine rupture at the exclusion of other much more common acute abdominal events in primigravid patients may be difficult, particularly during the first and second trimesters.

Uterine rupture in the second trimester, presenting with acute oligohydramnios as the first manifestation and in absence of other specific signs appears to be extraordinarily rare. To our knowledge, such a case has never been previously reported in the English or Chinese literature. Our patient initially presented to the local hospital, but they failed to determine the cause of the acute oligohydramnios. While the diagnosis of uterine rupture was eventually established in our hospital, the subtlety of preoperative symptoms may be explained by the intraoperative findings. The uterine tear was small, and only the increasing polyhydramnios together with evidence of ongoing bleeding raised suspicion of the diagnosis and prompted urgent abdominal exploration. Even though uterine rupture is rare, attention to even subtle signs and symptoms are critical to preserve maternal and fetal safety.

There are high-risk factors for uterine rupture of the gravid uterus in a primigravid woman, which include intrauterine surgery, oxytocin stimulation, placenta accreta, cocaine abuse, Ehlers-Danlos syndrome, in utero exposure to diethylstilbestrol, and uterine anomalies.^[9–16]^ A recent review including 36 published cases of uterine rupture in primigravid women identified the most common risk factor was prior uterine surgery, and less commonly, morbidly adherent placenta, congenital uterine anomaly, adenomyosis, connective tissue disorders, oxytocin, prostaglandin analogues, and labor.^[8]^ Moreover, a history of intrauterine manipulation, such as curettage and diagnostic hysteroscopy, can lead to an unrecognized uterine perforation.^[17]^ The present patients past medical history revealed no uterine operations, including intrauterine manipulation such as curettage. Abnormal placentation should also be considered a risk factor in the second trimester of pregnancy in patients who have no prior history of uterine instrumentation.^[18,19]^ This potential risk was excluded in the present patient by the normal placental location on repeated sonograms. Adenomyosis may be an uncommon risk factor for UR due to weakening of the uterine muscle.^[20–22]^ In our case, no evidence of pelvic endometriosis or adenomyosis was found during the operation. Moreover, the patient had no history of dysmenorrhea. Therefore, we were unable to identify a previously recognized risk factor for this patient, and, therefore, an exact cause of the UR remains unknown. In the English literature there are 4 reported cases of antepartum uterine rupture of the unscarred uterus in primigravid patients with no identified risk factors (Table 1).^[23–26]^ The most common rupture site proved to be the cornual area as it was in our case. Reportedly, such a spontaneous rupture of the unscarred gravid uterus, usually occurring in the cornual area, may be associated with focal weakness of the bilateral cornual uterine myometrium, and Müllerian duct anomalies related to the focal weakness.^[23,27]^ Therefore, although we could not definitively determine the cause of uterine rupture in this case, we could reasonably speculate that the primigravid patient in this case may have had focal weakness of the cornual uterine myometrium.

**Table 1:** Uterine rupture of the unscarred uterus in the primigravid patients with no identified high-risk factors.

Case Number	Age	Gravida-Para	Gestational age	Presenting symptom	Site of uterine rupture	Fetal outcome	Reference
1	29	G1P0	32 weeks	Abdominal pain	Right cornual area	Live birth	^[23]^
2	31	G1P0	21 weeks	Abdominal pain	Left cornual area	Live birth	^[24]^
3	20	G1P0	37 weeks	Abdominal pain	Left cornual area	Stillbirth	^[25]^
				Loss of fetal movement			
4	27	G1P0	32 weeks	Abdominal pain	Right uterosacral area	Live birth	^[26]^
				Nausea			

## 4 Conclusion

Clinical signs and symptoms of rupture of a gravid uterus in a primigravid patient are often nonspecific and can be confusing. It seems paradoxical that such a potentially catastrophic event of uterine rupture could present with only a single subtle manifestation and therefore pose such difficulty in being diagnosed early, but such can be the case, and it is important that obstetricians be reminded and cognizant of this dilemma, as it could well spell the difference between disaster and preserving maternal and fetal safety. Such increased awareness of spontaneous unscarred uterine rupture in a primigravid patient may prompt earlier diagnosis and implementation of immediate treatment. Therefore, we suggest that obstetricians should screen for high-risk factors for uterine rupture in the early stages of pregnancy. Obstetricians should consider the possibility of uterine rupture in pregnant females, even those without risk factors, in early gestational age when unexplained clinical manifestations, such as acute oligohydramnios, are discovered. Despite classic teaching that suggests the primigravid uterus is almost immune to spontaneous rupture,^[7,28]^ it should nevertheless be considered in the differential diagnosis when abdominal discomfort is accompanied by seroperitoneum.

## Author contributions

**Conceptualization:** Lingyun Yang.

**Data curation:** Lingyun Yang.

**Formal analysis:** Bo Zhang.

**Investigation:** Bo Zhang, Yifan Zhao, Chuan Xie.

**Methodology:** Yifan Zhao, Chuan Xie.

**Software:** Lingyun Yang, Bo Zhang, Chuan Xie.

**Supervision:** Lingyun Yang, Bo Zhang, Chuan Xie.

**Writing – original draft:** Lingyun Yang, Chuan Xie.

**Writing – review & editing:** Lingyun Yang, Chuan Xie.

## Abbreviations

Abbreviations: CS = cesarean section, UR = uterine rupture.

## References

[1] Hofmeyr GJ Say L Gulmezoglu AM. WHO systematic review of maternal mortality and morbidity: the prevalence of uterine rupture. BJOG 2005;112:1221–8. 16101600 10.1111/j.1471-0528.2005.00725.x

[2] Zwart JJ. Uterine rupture in the Netherlands: a nationwide population-based cohort study. BJOG 2009;116:1069–78. 19515148 10.1111/j.1471-0528.2009.02136.x

[3] Vernekar M Rajib R. Unscarred uterine rupture: a retrospective analysis. J Obstet Gynaecol 2016;66: (Suppl. 1): 51–4. 10.1007/s13224-015-0769-7 PMC5016409 27651577

[4] Ozdemir I Yucel N Yucel O. Rupture of the pregnant uterus: a 9-year review. Arch Gynecol Obstet 2005;272:229–31. 15843950 10.1007/s00404-005-0733-3

[5] Catanzarite V Cousins L Dowling D. Oxytocin-associated rupture of an unscarred uterus in a primigravida. Obstet Gynecol 2006;108:273–5. 17018478 10.1097/01.AOG.0000215559.21051.dc

[6] Mark B Landon. Uterine rupture in primigravid women. Obstet Gynecol 2006;108:709–10. 17018472 10.1097/01.AOG.0000236128.43970.aa

[7] Colin A Walsh Laxmi V Baxi. Rupture of the primigravid uterus: a review of the literature. Review Obstet Gynecol Surv 2007;62:327–34. 17425811 10.1097/01.ogx.0000261643.11301.56

[8] Walsh C Baxi L. Rupture of the primigravid uterus: a review of the literature. Obstet Gynecol 2007;62:327–34. 10.1097/01.ogx.0000261643.11301.56 17425811

[9] Saglamtas M Vicdan K Yalc¸in H. Rupture of the uterus. Int J Gynecol Obstetl 1995;49:9–15. 10.1016/0020-7292(95)02333-8 9457978

[10] Sun H Su W Chang W. Rupture of a pregnant unscarred uterus in an early secondary trimester: a case report and brief review. J Obstet Gynaecol Re 2012;38:442–5. 10.1111/j.1447-0756.2011.01723.x 22229814

[11] Martinez-Garza P Robles L Roca M. Spontaneous uterine rupture: report of two cases. Cir Cir 2012;80:78–82. 22472159

[12] Walsh C Reardon W Foley M. Unexplained prelabor uterine rupture in a term primigravida. Obstet Gynecol 2007;109:455. 10.1097/01.AOG.0000244699.66548.e3 17267858

[13] Pepin M Schwarze U Superti-Furga A. Clinical and genetic features of Ehlers-Danlos syndrome type IV, the vascular type. N Engl J Med 2000;342:673–80. 10706896 10.1056/NEJM200003093421001

[14] Agarwal R Gupta B Radhakrishnan G. Rupture of intrapartum unscarred uterus at the fundus: a complication of passive cocaine abuse? Arch Gynecol Obstet 2011;283:53–4. 10.1007/s00404-011-1853-6 21327801

[15] Porcu G Courbiere B Sakr R. Spontaneous rupture of a first-trimester gravid uterus in a woman exposed to diethylstilbestrol in utero: a case report. J Reprod Med 2003;48:744–6. 14562644

[16] Nishi H Funayama H Fukumine N. Rupture of pregnant noncommunicating rudimentary uterine horn with fetal salvage: a case report. Arch Gynecol Obstet 2003;268:224–6. 12942254 10.1007/s00404-002-0310-y

[17] Nkwabong E Kouam L Takang W. Spontaneous uterine rupture during pregnancy: case report and review of literature. Afr J Reprod Health 2007;11:107–12. 20690294

[18] Blè R Adjoussu K Doukoure SB. Placenta percreta: a rare etiology of spontaneous uterine perforation in the second trimester of pregnancy. Gynecol Obstet Fertil 2011;39:e11–4. 21183389 10.1016/j.gyobfe.2010.08.004

[19] Pistofidis G Makrakis E Balinakos P. Report of 7 uterine rupture cases after laparoscopy myomectomy: update of the literature. J Minim Invasive Gynecol 2012;19:762–7. 23084683 10.1016/j.jmig.2012.07.003

[20] Locci R Nisolle M Angioni S. Expression of the gamma 2 chain of laminin-332 in eutopic and ectopic endometrium of patient with endometriosis. Reprod Biol Endocrinol 2013;11:94. 24070183 10.1186/1477-7827-11-94 PMC3849601

[21] Nikolaou M Kourea H Antonopoulos P. Spontaneous uterine rupture in a primigravid woman in the early third trimester attributed to adenomyosis: a case report and review of the literature. J Obstet Gynecol Res 2013;39:727–32. 23151226 10.1111/j.1447-0756.2012.02042.x

[22] Pisanu A Deplano D Angioni S. Rectal perforation from endometriosis in pregnancy: case report and literature review. World J Gastroenterol 2010;16:648–51. 20128037 10.3748/wjg.v16.i5.648 PMC2816281

[23] Etsuko Mizutamari Tomoko Honda Takashi Ohba. Spontaneous rupture of an unscarred gravid uterus in a primigravid woman at 32 weeks of gestation. Case Rep Obstet Gynecol 2014;2014:209585. 25093133 10.1155/2014/209585 PMC4100263

[24] Wang P Chao H Too L. Primary repair of cornual rupture occurring at 21 weeks gestation and successful pregnancy outcome. Hum Reprod 1999;14:1894–5. 10402413 10.1093/humrep/14.7.1894

[25] Abbi M Misra R. Rupture of uterus in a primigravida prior to onset of labor. Int J Fertil Womens Med 1997;42:418–20. 9459086

[26] Langton J Fishwick K Kumar B. Spontaneous rupture of an unscarred gravid uterus at 32 weeks gestation. Hum Reprod 1997;12:2066–7. 9363731 10.1093/humrep/12.9.2066

[27] Leroux M Coatleven F Faure M. Bilateral uterine rupture of an unscarred gravid uterus before labor. Gynecol Obstet Fertil 2014;42:454–7. 24394323 10.1016/j.gyobfe.2013.08.018

[28] O’Driscoll K Meagher D Robson M. Active Management of Labor. 4th ed London: Mosby; 2003.

